# *Aedes albopictus* Sterile Male Production: Influence of Strains, Larval Diet and Mechanical Sexing Tools

**DOI:** 10.3390/insects13100899

**Published:** 2022-10-03

**Authors:** Marco Malfacini, Arianna Puggioli, Fabrizio Balestrino, Marco Carrieri, Maria Luisa Dindo, Romeo Bellini

**Affiliations:** 1 Department of Agricultural and Food Sciences, University of Bologna, Viale Fanin, 42, 40127 Bologna, Italy; 2 Department of Medical and Veterinary Entomology, Centro Agricoltura Ambiente “G. Nicoli”, Via Sant’Agata 835, 40014 Crevalcore, Italy

**Keywords:** sterile insect technique, *Aedes albopictus*, sex separation, mass rearing

## Abstract

**Simple Summary:**

*Aedes albopictus* is a vector able to transmit several arboviruses. Due to its high impact on human health, it is important to develop an efficient control strategy for this pest. Nowadays, control based on chemical insecticides is limited by the number of available active principles and the emergence of resistances. A valuable alternative to classic control strategies is the sterile insect technique (SIT) which relies on the release of the target insect sterile males. Mating between wild female and sterile male results in no viable offspring. A crucial aspect of SIT is the production of a large number of sterile males with a low presence of females that can bite and transmit viruses. Since productivity and female contamination during mass production are affected by different variables, in this study we investigated mechanical sorting tools, strain and larval diets. It emerged that the use of the sieve could be limited by colony adaptation over breeding generations, while the Fay-Morlan separator could be a valuable tool to overcome this issue. Strains with different degrees of colonization and larval diets affect the productivity and the female presence; therefore, control of these variables could improve the feasibility and reduce the costs of SIT programs.

**Abstract:**

The sterile insect technique (SIT) is a biologically based method of pest control, which relies on the mass production, sterilization, and release of sterile males of the target species. Since females can transmit viruses, it is important to develop a mass rearing system to produce a large number of males with a low presence of females. We evaluated the effects of different strains, larval diets and sexing tools on male productivity and residual female presence for the application of SIT against *Aedes albopictus*. Strains coming from Italy, Germany, Greece, and Montenegro, with different levels of colonization, were reared with three larval diets: IAEA-BY, BLP-B and SLP-BY. Developed pupae were sexed using two different mechanical methods: sieve or Fay-Morlan separator. The results proved that adoption of the Fay-Morlan separator increased the productivity and limited the female presence. The IAEA-BY diet showed the lowest female contamination. Strains with a high number of breeding generations showed a decreased productivity and an increased female presence. Increased female presence was found only in extensively reared strains and only when the sorting operation was conducted with sieves. We hypothesize that extensive colonization may determine a size reduction which limits the sexing tool efficiency itself.

## 1. Introduction

*Aedes albopictus* (Skuse), commonly known as the Asian tiger mosquito, is a mosquito species native to Southeast Asia that has spread to several continents, mainly due to the international used-tire trade [1,2,3]. This species has established itself in several countries [4], thanks to its high adaptive potential and the ability of its diapausing eggs to overcome winter seasons [5].

Being anthropophilic and feeding during daylight hours, this mosquito can strongly influence the use of green spaces, especially within urban contexts, which have the ideal conditions for its proliferation. The most essential aspect of *Ae. albopictus* is its ability to transmit viruses to vertebrates, including human beings, which is an element of considerable health importance. 

The main control strategy of *Ae. Albopictus* aims to reduce its field population density through insecticide application [6] using chemicals or microbial larvicide and growth regulator [7,8,9]. 

Mosquito control using insecticides, coupled with potential larval breeding site removal and information campaigns, gives only partially satisfactory results [10]. Furthermore, the increasing insecticide resistance is strongly affecting insecticide effectiveness [8,11]. For these reasons, further control techniques are needed. The integrated approach of conventional vector control and biocontrol could offer a valuable strategy to maintain and control mosquito population density. Traditional biocontrol approaches, such as larvivores fish and copepods, bacterial and fungal pathogens, and endosymbionts could be used [12,13]. Another interesting biocontrol approach could be the mosquito genetic control strategies (GCSs), that have become an important research area on account of their species-specificity, track record in targeting agricultural insect pests, and environmentally non-polluting nature [14]. 

Since 2000, research projects on the application and development of the sterile insect technique (SIT) on *Ae. albopictus* have been ongoing in Italy [15]. 

SIT involves the mass rearing, sterilization and systematic area-wide release of sterile males of a target pest, in this case *Ae. albopictus*. A sterile male mating with a wild type female results in no offspring, leading to population decline [16].

Sterile male mass production encompasses several steps to properly rear the insect, including sex separation, which is a crucial aspect. The presence of females in the SIT release campaigns must be avoided or strongly reduced. In fact, even if completely sterilized, the females maintain their feeding activity and vectorial capacity [17,18].

Sex separation of *Ae. albopictus* can be effectively achieved by exploiting different biological traits at either the pupal or adult stage, such as protandry [19] and dimorphism [20,21,22], or by investigating classical genetic or transgenic methods [14,23,24]. 

Classical genetic or transgenic methods are based on the establishment of genetic sexing strains (GSSs), which are laboratory strains of target insects whose genetics have been manipulated to allow efficient sex separation [14,25]. They can be created using classical mutagenesis to select a marker, such as a cuticle color [26], chemical or temperature sensitivity [27,28] to exclusively induce male production or by inducing an androgenization process [29,30]. GSSs have been generated in dozens of insect species, including mosquitoes, such as *Anopheles albimanus* [31], *Anopheles arabiensis* [32], *Ae. albopictus* [29] and *Aedes aegypti* [30], using insecticide resistance translocations that make males insensitive to the treatment [14,25] or transgenic expression of NIX locus [33,34]. However, environmental problems related to the use of insecticides, inefficient selection, low production and the need for a mother colony have so far prevented their development [32].

The difficulty in generating mutants using classical mutagenesis, in terms of time and laboratory activities, are shifting the focus on recombinant technologies. These systems are only used on a small-scale, and questions about their scalability, stability, accuracy, productivity and operating costs remain unanswered [14,25,35].

Even if the methods described allow effective sexing on a theoretical level, changes and improvements must be made in the perspective of mass production.

These methods have to comply with European regulations, which limits the use of genetic manipulation, and require that the breeding l for continuous production must be maintained [35].

Nowadays, the most employed sexing methods are based on mechanical sexing tools, which exploit protandry, according to which males develop earlier compared to females, and rely on sex size dimorphism present at the pupal stage, whereby females are bigger than males [15,24,36,37,38,39,40]. The developmental rates of males, females and larvae are not synchronous, and are time-related, determining the simultaneous presence of pupae of both sexes and larvae.

Mechanical sexing, and larval removal, can be performed using sieves, which are characterized by specific mesh sizes [15,21]. An alternative is the Fay-Morlan separator [20], with its adaptation [22] and mechanization [41,42].

These tools help to collect male pupae with the lowest number of females. The fixed mesh size of the sieve, the thickness adjustment of the Fay-Morlan device, the operating procedures, and the sample variability are delicate aspects that can affect their efficiency.

Sexing tools, used to separate immature stages with different degrees of development, can recover only part of all the developed males, since pupation dynamics is affected by rearing conditions.

During SIT programs, the maintenance of the breeding colonies could be based on different rearing routines and time schedules [43,44]. At the CAA laboratory, the individuals employed to sustain the breeding lines came from the discarded material of the sex sorting procedure, to better fit space and labor cost, without the necessity to maintain a mother colony.

The extensive rearing and selection process could lead to inbreeding and laboratory adaptations affecting the quality of the reared strains [45,46,47]. Strains, over generations, could vary their productivity affecting both male and female production.

The present study aims to find the most reliable mechanical sex sorter tool that presents the highest male productivity with the lowest female presence. We investigated the efficiency of sieves and Fay-Morlan separators as *Ae. albopictus* sexing tools and analyzed the effects of diets and strains. Relying only on the efficiency of the sorting tool, without considering other aspects, such as those associated with reared colonies, number of generations or diets, could strongly reduce the potential of the sorting tool.

The presented data were collected in the mosquito mass rearing laboratories at the Centro Agricoltura Ambiente “G. Nicoli” Srl (CAA) (Crevalcore, Italy) between 2018 and 2019. During this period, CAA mass-reared and supplied sterile males for SIT trials in Italy, Montenegro, Greece and Germany.

To prevent concerns about possible introduction of exotic genetic background, country-based colonies were initiated from field-collected eggs [48]. Strains were mass-reared to sustain male mass production for each SIT program.

During the period considered, each colony had different breeding generations and was reared on different larval diets. In particular, the IAEA-BY diet [49], a standard diet used for rearing, was compared with two other diets, based primarily on bovine or swine liver powder, due to poor availability and high costs of IAEA-BY diet ingredients.

As already observed by Puggioli and colleagues [50], diet may influence male productivity and sex ratio. Possible interaction of, or effect by, colonized strains has not been studied yet.

Strains from distinct countries and from different breeding generations could show variable productivity or residual female presence levels. In addition, a strain fed on a different diet could modify its own development, influencing the sex sorting operation.

## 2. Materials and Methods

### 2.1. MosquitoStrains and Colonization

*Ae. albopictus* strains were established via collecting eggs from fields in Italy, Germany, Montenegro, and Greece. Samples of a few thousand eggs were provided by the German Mosquito Control Association, the University of Montenegro, and the Benaki Phytopathological Institute.

Each strain was reared in 40 × 40 × 40 cm Plexiglass cages, under standard conditions at 28 ± 1°C, 85% RH, and a photoperiod of 14:10 (L:D) at the Centro Agricoltura Ambiente “G. Nicoli” Srl laboratory. Adults were constantly supplied with 10% sucrose solution, with the females offered swine blood daily through a thermostat-controlled device. Eggs laid on wet wrinkled paper were removed from the adult cages and placed, when dry, in sealed plastic boxes to maintain relative humidity close to 100% [51].

Eggs were counted using ImageJ software in order to precisely hatch the corresponding amount of L1 larvae for each rearing tray. The desired number of eggs was then put overnight in sealed containers with nutrient broth solution and brewer yeast to induce hatching, as reported by Balestrino et al., 2010 [52]. First instar larvae were transferred to 35 × 25 cm plastic trays at a density of 2 larvae/mL of deionized water [53] and fed with three different diets.

Between seasons of 2018 and 2019, four different colonies were used: the Italian strain (IT), native to Rimini, Italy; the Montenegrin strain (ME), native to Podgorica, Montenegro; the German strain (DE), native to Heidelberg, Germany; and the Greek strain (GR), native to Athens, Greece. All these colonies were continuously mass-reared with different breeding generations produced. In particular, the ME and the GR strains had less than eight breeding generations, the DE strain had between 9 and 18 generations, and the IT, which was the most extensively used strain, had more than 68 breeding generations.

In this study, we classified each strain into two main groups, based on the number of breeding generations. Strains with a low generation number (LGN), i.e., less than 18 generations, were represented by GR and ME, while the DE and IT strains had a high generation number (HGN).

### 2.2. Diet Preparation

The three diets used to evaluate the effects of larval food on the male productivity yield and the residual female presence were:

(1) IAEA-BY 5% wt:vol diet (developed at the FAO/IAEA Insect Pest Control Laboratory)—36% bovine liver powder (MP Biomedicals, Santa Ana, CA, USA), 50% tuna meal (T. C. Union Agrotech, Thailand), 14% brewer yeast (SigmaAldrich, St. Louis, MO, USA), and, as an additive, 0.2% *w*/*v* of Vitamin Mix (Vanderzant Vitamin Mix, Bio-Serv, Frenchtown, NJ, USA) [49,50,54].

(2) BLP-BY 5% wt:vol diet—86% bovine liver powder (MP Biomedicals, Santa Ana, CA, USA), 14% brewer yeast (SigmaAldrich, St. Louis, MO, USA), and, as an additive, 0.2% *w*/*v* of Vitamin Mix (Vanderzant Vitamin Mix, Bio-Serv, Frenchtown, NJ, USA).

(3) SLP-BY 5% wt:vol diet—86% swine liver powder (Mucedola Srl, Settimo Milanese, Milano, Italy), 14% brewer yeast (Mucedola Srl, Settimo Milanese, Milano, Italy), and, as an additive, 0.2% *w*/*v* of Vitamin Mix (Mucedola Srl, Settimo Milanese, Milano, Italy).

A slurry of each diet was prepared by manually mixing the solid components, consisting of small particles, in deionized water. Using a volumetric cylinder, diet was introduced in each rearing tray.

Since hatching, for the first four days, 0.2, 0.4, 0.6, and 0.8 mg/larval diet were daily provided for each rearing tray [51,53].

### 2.3. Mechanical Sex Sorting Tools and Procedures

Twenty-four hours from the onset of pupation, larvae and pupae reared on each diet were sexed using calibrated metal sieves and Fay-Morlan glass plate separators (Guangzhou Wolbaki Biotech Co. Ltd., Guangzhou, China) in order to assay their productivity and the residual female presence [15,20,21,22,51,55].

When sieve sorting was employed, the material collected from each tray was transferred into buckets filled with tap water at 34 °C. The set temperature encouraged the pupal emersion while larvae tended to accumulate in the basal part of the bucket. A sieve with a mesh size of 1400 µm was placed inside the bucket over the water surface, and left for 3 min, allowing male pupae to pass through the sieve net, while larvae and female pupae remained under the sieve, and a large number of small larvae were able to pass the sieve together with the male pupae. The males passed through the sieve were then collected [15,51,56], while larvae present between males were manually removed.

The Fay-Morlan separator (Guangzhou Wolbaki Biotech Co. Ltd., Guangzhou, China) is composed of two adjustable paired sheets of glass, and modifying the distance between the two sheets allows the separation of larvae, and male and female pupae by size. Samples were introduced in the upper part of the instrument and, through water washing, it was possible to spread the biological material through the glass plate. In accordance with the set distance, it was possible to differentiate and separately collect larvae, and male pupae and female pupae.

After the sorting procedures, male pupae were counted using a graduated cylinder with a net underneath to allow water drainage. The graduated cylinders were previously calibrated by counting and male samples from each reared strain were measured in order to avoid any possible influence due to dimensional differences in each strain.

### 2.4. Evaluated Parameters and Statistical Analysis

The effectiveness of different mechanical sex separation methods, according to the reared strains and used diets, was evaluated by the male productivity yield and the residual female presence together with the sorted males.

The productivity yield was calculated as the percentage of male pupae collected on the initial number of first instar larvae. In order to obtain the male productivity yield on the total number of reared males, results were multiplied by two, assuming an equal proportion of males and females on hatched L1, as also reported by Crawford et al., 2020 [57].

(1) $$Male\;productivity\;yield\;\%=\left[\frac{No.\;males}{No.larvae}\times 100\right]\times 2$$

*‘No.males’* is the number of male pupae after sex sorting, and *‘No.larvae’* is the number of larvae present in the trays at the beginning of rearing. The ratio of ‘*No.males*’ to ‘*No.larvae*’ represents the productivity of male pupae over the reared larvae.

Residual female presence was estimated by evaluating the sex ratio in a randomly collected sample of about 300 pupae in each batch of male pupae collected after each mechanical sorting session.

(2) $$Residual\;female\;presence\;\%=\left[\frac{No.\;females}{No.pupae}\times 100\right]$$

where *‘No.females’* is the number of female present in the sample and *‘No. pupae’* is the total number of pupae checked. The sex ratio observations were made by using a binocular stereomicroscope to analyze the tenth abdominal segment of each pupa [58]. The numbers of total pupae and females were counted separately. The number of females found was divided by the total number of pupae checked, and the result was multiplied by 100 to give the percentage of residual female *‘No.females’*.

In this study, the male productivity was corrected on the basis of the residual female presence to offer more truthful data on productivity as reported below:

(3) $$Corrected\;Productivity\;\%=\left[\left(1-\frac{R.\;f.\;presence}{100}\right)\times M.\;p.yield\right]$$

where *‘R. f. presence’* is the residual female presence and ‘*M. p. yield’* is the male productivity yield.

Two main analyses were performed using the R-based program Jamovi (version 2.2). The post hoc analyses were carried out using Tukey correction.

The first analysis aimed to investigate differences in male productive yield and residual female presence between 2018 and 2019 when using a sieve and a Fay-Morlan separator.

Two-way ANOVA analysis was carried out, with the male productivity yield and the residual female presence adopted separately as dependent variables, while reared strain and sexing tool were adopted as the fixed factors. The interaction effect between the fixed factors was also analyzed.

The second analysis studied the influence of three different larval diets and three strains on the male productivity yield and the residual female presence. In this case, the sorting procedure was carried out 24 h from the onset of pupation in the 2019 season using only the Fay-Morlan separator.

Two-way ANOVA analyses were carried out using, respectively, male productivity yields and residual female presence as dependent variables, while diet and strain were set up as fixed factors. The interaction effect of these factors was analyzed.

## 3. Results

### 3.1. Sexing Tool Comparison with Different Strains

The comparison of male productivity yields between strains and the sex sorting separator showed significative differences (F_3,53_ = 3.18, *p* < 0.05 and F_1,53_ = 5.75, *p* < 0.05), but their interaction effect was not significant (F_3,53_ = 1.88, *p* > 0.05).

When strains were compared pairwise a significant increase of 8.6 ± 3.19% was observed between ME and DE strains (t_53_ = 2.70, *p* < 0.05), but no significant differences were found between each other’s strain pairwise (*p* > 0.05).

With the Fay-Morlan separator the male productivity was 33.7 ± 2.02%, compared to 27.4 ± 1.68% for the sieve. Thus, the Fay-Morlan separator showed a mean significant improvement of 6.3 ± 2.63% (t_53_ = 2.4, *p* < 0.05).

Post hoc analysis between sexing tools for each reared strain showed a mean significant increase in male productivity of 15.6 ± 4.82% with the Fay-Morlan separator, compared to the sieve, for only the GR strain (t_53_ = 3.24, *p* < 0.05). Other strains showed no significant differences between each other pairwise (*p* > 0.05).

The productivity of all the reared strains showed no significant difference between the two different sexing methods (*p* > 0.05) (Table 1 and Table 2). Comparison of the residual female presence as a function of the strain and of the sexing tool showed a significant effect for the sexing tool variable (F_3,53_ = 70.79, *p* < 0.001).

The sieve method gave the highest residual female presence of 4.52 ± 0.29%, with a significant mean difference of 3.81 ± 0.05% compared to the Fay-Morlan separator (t_53_ = 8.41, *p* < 0.001), which gave a residual female presence of 0.71 ± 0.35%

The effect of the strains was also significant (F_3,53_ = 9.18, *p* < 0.001). In particular, the IT strain showed the highest mean level of residual female presence of 5.09 ± 0.59%, with significant differences between this strain and each of the other reared strains; it showed significant mean differences of 3.46 ± 0.72%, 3.25 ± 0.73% and 3.20 ± 0.68% compared to the GR, DE and ME strains (^MN^t_53_ = 4.72, ^MN^*p* < 0.001; ^DE^t_53_ = 4.45, ^DE^*p* < 0.001 and ^GR^t_53_ = 4.80, ^GR^*p* < 0.001). No significant effect was found between strains pairwise (*p* > 0.05). The effect of the interaction between strains and sexing tools was also significant (F_3,53_ = 10.38, *p* < 0.001), suggesting that the effect of a single variable was influenced by the effect of the other variable. Post hoc analysis of residual female presence between sexing tools for each reared strain showed significant differences only in the IT strain, with an increased mean difference of 8.99 ± 1.18% (t_53_ = 7.64, *p*_tukey_ < 0.001). No significant differences between sex sorters were observed in the MN, GR and DE strains (t_53_ = 2.45, *p* > 0.05, t_53_ = 2.59, *p* > 0.05 and t_53_ = 2.83, *p* > 0.05).

Post hoc analysis of strains sexed with a sieve or a Fay-Morlan separator showed significant differences between the IT strain and other strains only when a sieve was used. The IT strain sex-sorted with a sieve showed a higher mean value of 9.59 ± 0.77% of residual female presence, with significant mean differences of 6.52 ± 0.87%, 6.87 ± 0.89% and 6.87 ± 0.99% compared with the DE, ME and GR strains. No differences were found between strains when sex sorting was done using the Fay-Morlan separator (*p* > 0.05) (Table 1, Table 2 and Table S1).

### 3.2. Larval Diet and Strain Influence with the Fay-Morlan Separator

The effects of strains and diets on male productivity yield were significant (F_2,45_ = 10.38, *p* < 0.001 and F_2,45_ = 8.85, *p* < 0.001), while the effect of their interaction was not significant (F_4,45_ = 0.43, *p* > 0.05).

The GR F5-F8 strain showed the highest productivity of all the strains, with a mean male productive yield of 39.7 ± 1.79%. The productivity of this strain was 11.9 ± 2.96% higher in comparison with IT F73-F74 (t_45_ = 4.01 *p* < 0.001) and 10.0 ± 2.82% higher with respect to the DE F16-F18 strain (t_45_ = 3.54, *p* < 0.01). No significant difference was found between DE F16-F18 and IT F73-F74 (t_45_ = 0.58, *p* > 0.05). The male productivity yield was also influenced by larval diet, with a significant difference of 11.7 ± 2.78% between SLP-BY and BLP-BY (t_45_ = 4.21, *p* < 0.001). The BLP-BY diet showed the highest male productivity yield of 38.0 ± 1.88%. The differences between the IAEA-BY diet and either of the BLP-BY and SLP-BY diets were not significant (t_45_ = 1.71, *p* > 0.05 and t_45_ = 2.05, *p* > 0.05 respectively).

Post hoc analysis of the three diets for each reared strain showed no significant difference (*p* > 0.05). Interestingly, the mean values and data distribution of the male productive yield as a function of the diet followed similar trends in all reared strains.

Comparison of the productivity of each strain reared on the three diets showed no significant difference (*p* > 0.05) (Table 3 and Table 4). The residual presence of females was influenced by diets (F2,45 = 13.58, *p* < 0.001), but the strains showed no significant effect (F2,45 = 1.99, *p* > 0.05). An interaction effect between these two variables was not found (F4,45 = 0.73, *p* > 0.05).

The IAEA-BY diet gave the lowest value of residual female presence of 0.59 ± 0.34%. There were significant mean differences of 1.83 ± 0.43% between IAEA-BY and BLP-BY and of 1.3 ± 0.45% between IAEA-BY and SLP-BY (t45 = 5.13, *p* < 0.001 and t_45_ = 3.80, *p* = 0.001). No difference was found between BLP-BY and SLP-BY (t45 = 1.33, *p* > 0.05). Post hoc analysis comparing residual female presence values obtained using the three diets for each reared strain showed no significant differences (*p* > 0.05). As already observed for male productivity yield, the distribution of residual female presence showed the same trends for each strain according to the diet.

The residual female presence between diets according to the strain showed no significant differences (Table 3, Table 4 and Table S1).

## 4. Discussion

Mass production of sterile male mosquitoes is a complex activity strictly connected to larval rearing, which is affected by different variables, such as genetic background, synchronous growth, temperature, humidity, larval density and diets [40,59,60,61]. The capacity to collect large quantities of males with the lowest residual number of females strongly affects the feasibility of SIT application [51,62,63,64].

The Fay-Morlan separator represents a better alternative to the sieve as the mechanical sexing tool. The residual female presence, obtained by the use of the Fay-Morlan separator, showed similar values to other SIT trials carried out by Zheng et al., 2019 in *Ae albopictus* [65] and by Carvalho et al., 2014 in *Ae. aegypti* [66], using the same sorting method.

Besides the improved efficiency of the Fay-Morlan sexing separation for both productivity and female presence, there was reduced labor time and improved management which led to a cost–benefit advantage for the whole production [43]. The operating procedures connected to the use of sieves are highly time-consuming due to the management of the biological material [14,25]. Indeed, the sieving method is quite laborious, with sample material from each rearing tray needing to be sieved into buckets filled with water at 34 ± 1 °C [15]. The sieve must then be placed in the upper part of the bucket and left for 3 min to allow male emergence through the mesh [21], and larvae still present between males have to be manually removed or treated with *Bacillus thuringiensis.* The treatment with *B. thuringiensis*, which leads to larval death without affecting pupae survival, may still determine a contamination of the larval rearing with consequent potential risks. Pupae that passed through the mesh need to be transferred to dedicated containers for final counting and sex ratio evaluation. Material unable to pass through the sieve, comprised of larvae and female pupae, and some male pupae, must be collected and replaced with new material. Furthermore, during the whole sieving activity, water temperature must always be regulated to encourage male emergence [15]. In addition, it is not possible to regulate the sieve mesh size to prevent female contaminations unless using a different fixed mesh size sieve. The Fay-Morlan separator has the distinct advantage of removing larvae from the collecting of pupae.

The Fay-Morlan separator has made it possible to consecutively remove larvae and male and female pupae in one step [20,22]. After the introduction of biological material, water is drained into the separator to allow sample dispersion over the glass plate. Through an initial regulation of the upper screws, it is possible to use only the bottom screws of the separator during the sorting operation, so, adjusting the distances between the sheets of glass, pupae and larvae are able to flow out easily through the separator step by step. It is important for the operator to adjust the distances between the sheets according to the pupal distribution and size dimorphism to prevent female contamination [22]. Material could be directly collected in dedicated trays for larvae, and male pupae and female pupae, that are directly placed underneath the Fay-Morlan separator.

Even if the Fay-Morlan separator is a better alternative to the sieve, the operating procedure connected with the use of this tool is highly repetitive and demanding for the operators’ wellbeing, especially for continuous and prolonged periods [14]. Furthermore, the residual female presence is highly dependent on the expertise of the operator and how the separation is carried out. Selection of smaller individuals reduces female contamination but, at the same time, results in loss of a larger number of males [67], so affecting the male productivity.

Automation of sex sorting procedures and equipment can reduce labor costs and other issues, such as human error and microbial contamination, and boost space utilization efficiency [43]. An automatic sex sorter based on a robotized Fay-Morlan separator has already been developed by Wolbaki Company [42], and some efforts on computer vision have also been conducted to separate males based on pupal [68] and adult dimorphism [57].

Proper automation of the Fay-Morlan tool could offer a great advantage for extensive SIT programs, but inherent limitations of exploiting sex size dimorphism mean that optimal results may not be achieved, since mass production is also affected by a number of other variables [50,56,60,61].

This study demonstrated how the use of different strains, in combination with different mechanical sexing tools, influenced the male productivity yield and residual female presence. It is possible that strains with different numbers of breeding generations (low colonization versus high colonization) could have different effects when sex-sorted with a specific sorting tool.

As reported by Hendrichs and Robinson, 2021, mass rearing protocols for SIT are focused on producing males for field release. Using the material recollected after the mass rearing permits the maintenance of the colony, but with this system it is impossible to avoid highly selected genotype accumulation [69]. In fact, under extensive breeding, modification of reproduction, development, and courtship behavior has already been observed [62,70].

Strains with low generation numbers, i.e., the ME and GR strains, showed higher mean male productivity yields than the DE and IT strains with high generation numbers. An extensively reared strain, for instance, the IT strain, presented a significant increased level of residual female presence with respect to all the other strains.

The increased level of female residual presence in the IT strain when sex-sorted with the sieve suggests that females may become smaller over generations, enabling them to pass through the fixed mesh size of 1400 µm of the sieve.

The extensive breeding procedure (high colonization) might have influenced both the male productive yield and the residual female presence. It is plausible that the number of breeding generations of the reared colony is associated with the size variations, which affects the productivity yield and female presence once sex-sorted. Size fluctuations have already been observed in adults held in cages over generations [36,71], as well as in pupae when the strain was maintained or the strains were cross-bred [19,72,73].

Body size adaptation over generations, during mass rearing, could not exclude a loss of range dimensional dimorphism between males and females. Thus, using a mechanical sexing system with a fixed mesh size, such as the sieve, could affect the male productivity and residual female presence, but the Fay-Morlan device can be adjusted to better fit the pupal dimensions [20,22], overcoming size-related problems to adaptation of the colony to cage breeding.

The correlation between pupal and adult body sizes in both sexes have already been already [74]. In addition, size differences in adults have been reported to influence productivity, mating competitiveness, survival, dispersal rate, fecundity and egg production [36,71,75,76,77,78,79]. Furthermore, the effect of strain colonization on vector competence has been observed [46,47]. This suggests that not considering the possible variations in pupal size caused by different strains and extensive breeding processes could strongly affect the quantity and quality of male production [16,44,48].

The significant effects of the strains, also reared on different diets, on male productive yields confirmed our observation of the negative effect of continuous breeding in artificial conditions on sex separation outcomes. The GR strain, which had a low number of rearing generations, showed the highest male productive yield in comparison with the other reared strains. Differences in productivity between these strains could not be attributed with certainty to the number of breeding generations, but the absence of any significant difference between the MN and GR strains reared on IAEA-BY larval diet, which had similar ranges of breeding generations, suggests that a genetic feature specific to the MN strain might be missing, thus leading to increased productivity.

The absence of differences between strains, when considering the residual female presence, suggests that the Fay-Morlan separator was highly reliable in all strains. This also indicated that the decreased number of males obtained by continuous breeding could be attributed not only to a decreased pupation rate, but also to size dimorphism loss. To achieve lower levels of female contamination, operators may have to increase selectivity using the Fay-Morlan separator, thus reducing also the male recovery rate and compensating for differences in residual female presence between strains [67].

Among larval diets, the BLP-BY diet gave a high productivity value, suggesting a high capacity to promote pupation dynamics in the reared strain compared to the SLP-BY. The IAEA-BY diet showed an intermediate tendency to promote pupation dynamics with respect to the other two diets, even if this was not supported by statistical analysis. Larvae fed with bovine liver powder, which is in both BLP-BY and IAEA-BY diets, probably reached the critical weight needed for pupae formation earlier [50,80].

It has to be mentioned that all the strains, before the diet test, were reared on IAEA-BY diet and, thus, the possibility of some level of strain adaptation could not be excluded. Even in the absence of interaction between strains with different generation numbers, diet suggests a lack of strain adaptation to diet.

The results given by the BLP-BY diet indicated that varying only a specific diet content and its composition could modify the effect of the diet on male productivity.

The larval diets also influenced the residual female presence, with the IAEA-BY diet, which gave the lowest contamination level, confirming that different diets influence the residual female levels differently [49,50,61,81].

Different compositions of the diets may influence larval development, synchronous growth and size dimorphism. In fact, high values of residual female presence given by BLP-BY and SLP-BY diets did not necessarily amount to increased male productivity yields. This suggests that high residual female presence does not always correspond to an increased male productivity yield, with other factors besides speed of development probably influencing female contamination and lack of carbohydrates affecting larval development [50,80,82]. Without enough carbohydrates, the female larvae may develop into small-sized pupae, thus affecting the efficiency of sexing methods that cannot perfectly discriminate sexes based on size dimorphism.

In conclusion, the decreased productivity of highly colonized strains may be due to the adopted breeding scheme, which is determined by the need to collect pupating females one day from the onset of pupation [69,70].

The Fay-Morlan separator allows the maintenance of similar levels of residual female presence for all reared strains and larval diet, resulting in reliable and properly managed sex sorting. The number of generations and laboratory involuntary cross-breeding selection must be controlled, particularly in places where SIT programs are to last for many years [69].

To better understand the male productivity of extensively colonized strains, it would be necessary to also collect data on female productivity and its sex ratio. Understanding the overall number of males and females would make it possible to better clarify male pupae development and male recovery yield when the sorting is conducted. In fact, the number of females at the sorting time may be not sufficient to sustain the breeding lines, forcing the re-entry of discarded larvae into the rearing, leading to an increased labor cost. An alternative may be the identification of a specific sorting time, higher with respect to the twenty-four hours from the beginning of pupation, that would provide good productivity with low residual female presence, but at the same time enough females to sustain the adult rearing.

In addition, male and female pupal size assessment along generation, may provide a quality measure to assess strain adaptation. Since adult body size affects adult quality parameters, the pupal body size measure could also provide an important tool to measure the quality of adult males.

Even if in our study the IAEA-BY diet gave the best results, in terms of male productivity and residual female presence, the adoption of a cheaper diet remains crucial. The Black soldier fly larval powder, which was used by Mamai et al., 2019 [81], shows promising results and could ultimately reduce diet costs. Further studies on pupation dynamics, body size dimension and genetic background of both males and females in a mass rearing context and in the use of other dietary components are ongoing.

## Figures and Tables

**Table 1:** Male productivity yields and residual female percentages obtained with the two sexing tools for each strain.

			MPY		RFP	
STRAIN	N	♀/♂ TOOL	Mean ± SE (♀/♂ TOOL)	Mean ± SE (STRAIN)	Mean ± SE (♀/♂ TOOL)	Mean ± SE (STRAIN)
DE F9-18	4	Fay-Morlan	26.8 ± 4.46 ^a^	26.4 ± 2.51 ^b^	0.62 ± 0.77 ^a^	1.84 ± 0.43 ^a^
	15	Sieve	26.1 ± 2.30 ^a^		3.07 ± 0.40 ^a^	
GR F1-8	8	Fay-Morlan	41.4 ± 3.16 ^b^	33.6 ± 2.41 ^ab^	0.56 ± 0.54 ^a^	1.64 ± 0.42 ^a^
	6	Sieve	25.8 ± 3.64 ^a^		2.72 ± 0.63 ^a^	
IT F68-74	3	Fay-Morlan	30.2 ± 5.15 ^a^	27.3 ± 3.41 ^ab^	0.60 ± 0.89 ^b^	5.09 ± 0.59 ^b^
	4	Sieve	24.4 ± 4.46 ^a^		9.59 ± 0.77 ^a^	
ME F2-8	9	Fay-Morlan	36.6 ± 2.98 ^a^	35.0 ± 1.97 ^a^	1.06 ± 0.51 ^a^	1.89 ± 0.34 ^a^
	12	Sieve	33.5 ± 2.58 ^a^		2.72 ± 0.45 ^a^	

‘STRAIN’ indicates the origin of the strain and ‘F’ is the range of number of breeding generations. ‘N’ represents the replicates number. ‘♀/♂ TOOL’ indicates the mechanical sexing tool adopted. ‘MPY’ is the male productivity yield percentage. ‘RFP’ is the residual female presence percentage. ‘Mean ± SE (♀/♂ TOOL)’ is the marginal mean ± standard error of the values obtained with the two sexing tools for each strain. ‘Mean ± SE (STRAIN)’ is the marginal mean ± standard error of the grouped values obtained with the two sexing tools for each strain. Different superscript letters within a column indicate statistical differences *p* ≤ 0.05, Tukey’s mean separation test.

**Table 2:** Male productivity yields and residual female percentages for each reared strain for each sexing tool.

			MPY		RFP	
♀/♂ TOOL	N	STRAIN	Mean ± SE (STRAIN)	Mean ± SE (♀/♂ TOOL)	Mean ± SE (STRAIN)	Mean ± SE (♀/♂ TOOL)
Fay-Morlan	9	ME F2-8	36.6 ± 2.98 ^a^	33.7 ± 2.02 ^b^	1.06 ± 0.51 ^a^	0.71 ± 0.35 ^b^
	8	GR F1-8	41.4 ± 3.16 ^a^		0.56 ± 0.54 ^a^	
	3	IT F68-74	30.2 ± 5.15 ^a^		0.60 ± 0.89 ^a^	
	4	DE F9-18	26.8 ± 4.46 ^a^		0.62 ± 0.77 ^a^	
Sieve	12	ME F2-8	33.5 ± 2.58 ^a^	27.4 ± 1.68 ^a^	2.72 ± 0.45 ^a^	4.52 ± 0.29 ^a^
	6	GR F1-8	25.8 ± 3.64 ^a^		2.72 ± 0.63 ^a^	
	4	IT F68-74	24.4 ± 4.46 ^a^		9.59 ± 0.77 ^b^	
	15	DE F9-18	26.1 ± 2.30 ^a^		3.07 ± 0.40 ^a^	

‘♀/♂ TOOL’ indicates the mechanical sexing tool adopted. ‘N’ represents the replicates number. ‘STRAIN’ indicates the origin of the strain and ‘F’ is the range of number of breeding generations. ‘MPY’ is the male productivity yield percentage. ‘RFP’ is the residual female presence percentage. ‘Mean ± SE (STRAIN)’ is the marginal mean ± standard error of the values obtained with the reared strain for each sexing tool. ‘Mean ± SE (♀/♂ TOOL)’ is the marginal mean ± standard error of the grouped values obtained with all strains for each sexing tool. Different superscript letters within a column indicate statistical differences *p* ≤ 0.05, Tukey’s mean separation test.

**Table 3:** Male productivity yields and residual female percentages obtained with the three larval diets for each strain.

			MPY		RFP	
STRAIN	N	DIET	Mean ± SE (DIET)	Mean ± SE (STRAIN)	Mean ± SE (DIET)	Mean ± SE (STRAIN)
DE F16-18	6	BLP-BY	36.9 ± 3.47 ^a^	29.7 ± 2.18 ^a^	2.19 ± 0.49 ^a^	1.29 ± 0.31 ^a^
	4	IAEA-BY	26.8 ± 5.83 ^a^		0.62 ± 0.61 ^a^	
	6	SLP-BY	25.3 ± 3.01 ^a^		1.08 ± 0.49 ^a^	
GR F5-8	8	BLP-BY	45.3 ± 3.12 ^a^	39.7 ± 1.79 ^b^	2.01 ± 0.43 ^a^	1.44 ± 0.25 ^a^
	8	IAEA-BY	41.4 ± 2.89 ^a^		0.56 ± 0.43 ^a^	
	7	SLP-BY	32.3 ± 2.61 ^a^		1.76 ± 0.46 ^a^	
IT F73-74	7	BLP-BY	31.8 ± 4.17 ^a^	27.8 ± 2.35 ^a^	3.07 ± 0.46 ^a^	2.17 ± 0.33 ^a^
	3	IAEA-BY	30.2 ± 3.91 ^a^		0.60 ± 0.70 ^a^	
	5	SLP-BY	21.4 ± 2.70 ^a^		2.85 ± 0.54 ^a^	

‘STRAIN’ indicates the origin of the strain and ‘F’ is the range of number of breeding generations. ‘N’ represents the replicates number. ‘DIET’ indicates the larval diet used. ‘MPY’ is the male productivity yield percentage. ‘RFP’ is the residual female presence percentage. ‘Mean ± SE (DIET)’ is the marginal mean ± standard error of the values obtained with the three larval diet for each strain. ‘Mean ± SE (STRAIN)’ is the marginal mean ± standard error of the grouped values obtained with the three larval diets for each strain. Different superscript letters within a column indicate statistical differences *p* ≤ 0.05, Tukey’s mean separation test.

**Table 4:** Male productivity yields and residual female percentages obtained with the three reared strains for each larval diet.

			MPY		RFP	
DIET	N	STRAIN	Mean ± SE (STRAIN)	Mean ± SE (DIET)	Mean ± SE (STRAIN)	Mean ± SE (DIET)
BLP-BY	8	GR F5-8	45.3 ± 3.03 ^a^	38.0 ± 1.88 ^b^	2.01 ± 0.43 ^a^	2.42 ± 0.27 ^a^
	6	DE F16-18	36.9 ± 3.50 ^a^		2.19 ± 0.49 ^a^	
	7	IT F73-74	31.8 ± 3.24 ^a^		3.07 ± 0.46 ^a^	
IAEA-BY	8	GR F5-8	41.4 ± 3.03 ^a^	32.8 ± 2.41 ^ab^	0.56 ± 0.43 ^a^	0.59 ± 0.34 ^b^
	4	DE F16-18	26.8 ± 4.29 ^a^		0.62 ± 0.61 ^a^	
	3	IT F73-74	30.2 ± 4.95 ^a^		0.60 ± 0.70 ^a^	
SLP-BY	7	GR F5-8	32.3 ± 3.24 ^a^	26.3 ± 2.04 ^a^	1.76 ± 0.46 ^a^	1.90 ± 0.29 ^a^
	6	DE F16-18	25.3 ± 3.50 ^a^		1.08 ± 0.49 ^a^	
	5	IT F73-74	21.4 ± 3.84 ^a^		2.85 ± 0.54 ^a^	

‘STRAIN’ indicates the origin of the strain and ‘F’ is the range of number of breeding generations. ‘N’ represents the replicates number. ‘DIET’ indicates the larval diet used. ‘MPY’ is the male productivity yield percentage. ‘RFP’ is the residual female presence percentage. ‘Mean ± SE (STRAIN)’ is the marginal mean ± standard error of the values obtained with the three strains for each larval diet. ‘Mean ± SE (DIET)’ is the marginal mean ± standard error of the grouped values obtained with the three strains for each larval diet. Different superscript letters within a column indicate statistical differences *p* ≤ 0.05, Tukey’s mean separation test.

## Data Availability

Data is contained within the article or Supplementary Material. The data presented in this study are available in Supplementary Table S1.

## Supplementary Materials

The following are available online at https://www.mdpi.com/article/10.3390/insects13100899/s1, Table S1: Aedes albopictus Sterile Male Production: Dataset.
